# Supplementary material for: Smart polarization and spectroscopic holography for real-time microplastics identification
Source: Commun Eng. 2024 Feb 17;3:32. doi: 10.1038/s44172-024-00178-4 (PMC10955970; doi:10.1038/s44172-024-00178-4)
Supplement: Supplementary file 2 — Supplementary Information file [file 44172_2024_178_MOESM2_ESM.pdf]

Smart polarization and spectroscopic holography for real-time microplastics identification

Yanmin Zhu[1], Yuxing Li[1], Jianqing Huang[1,2], and Edmund Y. Lam[1,*]
[1]*Department of Electrical and Electronic Engineering, The University of Hong Kong, Pokfulam, Hong Kong SAR, China*
[2]*Key Lab of Education Ministry for Power Machinery and Engineering, School of Mechanical Engineering, Shanghai Jiao Tong University, 800 Dongchuan Road, Shanghai, 200240, China*
**e-mail: elam@eee.hku.hk*

Supplement Note S1

Table S1 NDTDM notations.

Notation	Description
G_h	Highest gray tone value in the image.
ε	A small number
$p_i = \frac{N_i}{n^2}$	The probability of occurrence of gray tone value i in an $N \times N$ image where $n = N - 2d$
N_g	Total number of different gray levels present in the image.

Supplement Note S2

Texture feature calculation

Neighbourhood Gray Tone Difference Matrix (NDTDM) [1] are texture visual features and calculated as follows.

Define $f(x, y)$ as the gray tone of a pixel at (x, y) and has gray tone value i. The average gray tone over a neighborhood centered at (x, y), but not included, is calculated as

$$A_i = A(x, y) = \frac{1}{W-1} \sum_{x'=-d}^{d} \sum_{y'=-d}^{d} f(x + x', y + y'), \text{ (s1)}$$

where $(x', y') \neq (0,0)$, d specifies the neighbourhood size and $W = (2d + 1)^2$. The ith entry in the NDTDM is

$$s(i) = \begin{cases} \sum |i - A_i|, \; for\ i \in N_i\ if\ N_i \neq 0 \\ 0, \; otherwise \end{cases} \text{, (s2)}$$

where $\{N_i\}$ is the set of all pixels having gray tone i.

Detailed NDTDM is defined as

$$Coarseness = \left[\varepsilon + \sum_{i=0}^{G_h} p_i s(i)\right]^{-1}. \text{ (s3)}$$

$$Contrast = \left[\frac{1}{N_g(N_g-1)}\sum_{i=0}^{G_h}\sum_{j=0}^{G_h} p_i p_j (i-j)^2\right]\left[\frac{1}{n^2}\sum_{i=0}^{G_h} s(i)\right]. \text{ (s4)}$$

$$Busyness = \frac{\left[\sum_{i=0}^{G_h} p_i s(i)\right]}{\left[\sum_{i=0}^{G_h} ip_i - jp_i\right]}, p_i \neq 0, p_j \neq 0. \text{ (s5)}$$

$$Complexity = \sum_{i=0}^{G_h}\sum_{j=0}^{G_h}\left\{\frac{|i-j|}{n^2(p_i+p_j)}\right\}\{p_i s(i) + p_j s(j)\}, p_i \neq 0, p_j \neq 0. \text{ (s6)}$$

$$Strength = \frac{\left[\sum_{i=0}^{G_h}\sum_{j=0}^{G_h}(p_i+p_j)(i-j)^2\right]}{\left[\varepsilon+\sum_{i=0}^{G_h} s(i)\right]}, p_i \neq 0, p_j \neq 0. \text{ (s7)}$$

Supplement Note S3

Fourier Power Spectrum (FPS) features

The FPS features are calculated by the Fourier transform of an image $f(x,y)$ [2, 3]. Fourier transform of $f(x,y)$ is defined by

$$F(u,v) = \int_{-\infty}^{\infty}\int_{-\infty}^{\infty} e^{-2\pi i(ux+vy)} f(x,y)dxdy, \text{ (s8)}$$

and the FPS is defined as $|F|^2 = FF^*$, where * denotes the complex conjugate.

The radial distribution of values in $|F|^2$ changes with texture coarseness. A coarse texture will have high values of $|F|^2$ concentrated near the origin. A fine texture will be spread out. Features with the following form helps for the feature analysis, denoted as

$$\varphi_r = \int_0^{2\pi} |F(r,\theta)|^2 \, d\theta, \text{ (s9)}$$

where r represents for the ring radius.

Angular distribution of values in $|F|^2$ changes with the directionality of the texture. An edge texture and lines in each direction θ will have high values of $|F|^2$ around $\theta + \left(\frac{\pi}{2}\right)$.

Features with an average of $|F|^2$ taken over a wedge-shaped region entered at the origin and follow the form as

$$\varphi_\theta = \int_0^{\infty} |F(r,\theta)|^2 d\theta, \text{ (s10)}$$

where θ is the wedge slope, are good for feature analysis.

For $N \times N$ image, FPS is defined as

$$F(u,v) = \frac{1}{N^2}\sum_{i,j=0}^{N-1} f(i,j)e^{-2\pi\sqrt{-1}(iu+jv)}, \text{ (s11)}$$

Where $0 \leq u$ and $v \leq N-1$.

Feature sets with a ring-shaped FPS have the form of

$$\varphi_{r_1,r_2} = \sum_{r_1 \leq u^2+v^2 \leq r_2} |F(u,v)|^2, \text{ (s12)}$$

with inner ring radii r_1 and outer ring radii r_2.
Features with a wedge-shaped FPS have the form of

$$\varphi_{\theta_1,\theta_2} = \sum_{\theta_1 \leq \tan^{-1}\left(\frac{v}{u}\right) \leq \theta_2} |F(u,v)|^2, \text{ (s13)}$$

DC terms are omitted in the calculations as it is common to all the wedges.

Supplement Note S4
Polarization and holographic features
Describe the full polarization states with Stokes vectors,

$$S_0 = \frac{1}{2}(I_0 + I_{45} + I_{90} + I_{135}), \text{ (s14)}$$
$$S_1 = I_0 - I_{90}, \text{ (s15)}$$
$$S_2 = I_{45} - I_{135}, \text{ (s16)}$$

where I_0, I_{45}, I_{90} and I_{135} are the intensity image under $0°$, $45°$, $90°$ and $135°$

Angle of polarization:

$$AoP = \frac{1}{2}\tan^{-1}(S_2/S_1). \text{ (s17)}$$

Degree of linear polarization:

$$DoLP = \frac{\sqrt{S_1^2+S_2^2}}{S_0}, \text{ (s18)}$$

To clearly describe the phase retardation and optical axis orientation, we define two auxiliary variables,

$\varepsilon_1 = \frac{I_{90} - I_0}{I_{90} + I_0}$, (s19)

$\varepsilon_2 = \frac{I_{45} - I_{135}}{I_{45} + I_{135}}$. (s20)

Phase retardation:

$Phase\ retardation = \sin^{-1}\sqrt{\varepsilon_1^2 + \varepsilon_2^2}$, (s21)

Optical axis orientation:

$Optical\ axis\ orientation = \frac{1}{2}\tan^{-1}({}^{\varepsilon_1}/_{\varepsilon_2})$, (s22)

Fringes contrast:

$Fringe\ contrast = (I_{max} - I_{min})/(I_{max} + I_{min})$, (s23)

where I_{max} and I_{min} are the maximum and minimum intensity value of the holographic image.

$Transparency = \frac{(255 - \bar{I})}{255} \times 100$, (s24)

where $\bar{I}$ is the average intensity value of the image.

Supplement Note S5
AUC calculation

First, calculate the true positive rate (TPR) and the false positive rate (FPR) of a classifier:

$TPR = \frac{TP}{TP + FN}$, (s25)

$FPR = \frac{FP}{FP + TN}$, (s26)

where TP represents the true positive sample, FP represents the false positive sample, TN represents the true negative sample, and FN represents the false negative sample.

$AUC = \int TPR \, d(FPR)$. (s27)

Supplementary References

[1] M. Amadasun and R. King, "Textural features corresponding to textural properties," *IEEE Trans. Syst. Man Cybern.,* vol. 19, pp. 1264-1274, 1989.

[2] J. S. Weszka, C. R. Dyer and A. Rosenfeld, "A Comparative Study of Texture Measures for Terrain Classification," *IEEE Transactions on Systems, Man, and Cybernetics,* Vols. SMC-6, pp. 269-285, 1976.

[3] C.-M. Wu, Y.-C. Chen and K.-S. Hsieh, "Texture features for classification of ultrasonic liver images," *IEEE Transactions on Medical Imaging,* vol. 11, pp. 141-152, 1992.
